# IMPACT OF SOCIAL SUPPORT AND LIVING ARRANGEMENT ON CAREGIVERS: ADDRESSING LONELINESS THROUGH A SUPPORTIVE NETWORK

**DOI:** 10.1093/geroni/igae098.0828

**Published:** 2024-12-31

**Authors:** Brianna Garrison

**Affiliations:** Southern Connecticut State University, New Haven, Connecticut, United States

## Abstract

This presentation aims to examine the relationship between caregiver of persons living with dementia’s loneliness, living arrangement, and social support. Chronic progressive diseases like dementia can be risk factors for loneliness and social isolation (National Academy of Sciences, Engineering, & Medicine, 2020). Caregivers of persons living with dementia are at greater risk of loneliness throughout the trajectory of their caregiving role (Adams, 2008; Moyle et al., 2011). Living arrangement is a social environment factor shown to impact loneliness in those living with dementia (Cohen-Mansfield et al., 2015; Shiells et al., 2019), but a dearth of literature exists exploring the effects on the caregiver. Lack of quality social support can influence the experience of loneliness of both caregivers and persons living with dementia (Chatters et al., 2018; Cohen-Mansfield et al., 2016; Pinquart & Sorenson, 2001). This mixed methods study of caregivers of persons living with dementia (N = 158) explored the impact of living arrangement on the loneliness of the caregiver and how social support may impact the relationship between these two variables. This study found social support is a significant predictor of reducing loneliness among caregivers for persons living with dementia in all living arrangements. In the qualitative interviews (N = 22), caregivers reported social support impacted their loneliness throughout their care recipients’ disease trajectory. This study highlights the importance of understanding the unique physical and social environmental factors that may influence caregivers of persons living with dementia’s experience of loneliness and how quality social support may decrease the impact.

